# Enzymatic Degradation of Poly(butylene succinate) Copolyesters Synthesized with the Use of *Candida antarctica* Lipase B

**DOI:** 10.3390/polym10060688

**Published:** 2018-06-20

**Authors:** Aleksandra Wcisłek, Agueda Sonseca Olalla, Andrew McClain, Agnieszka Piegat, Peter Sobolewski, Judit Puskas, Miroslawa El Fray

**Affiliations:** 1 Division of Functional Materials and Biomaterials, Faculty of Chemical Technology and Engineering, West Pomeranian University of Technology, Szczecin, Al. Piastow 45, 71-311 Szczecin, Poland; ka38103@zut.edu.pl (A.W.); agueda.Sonseca-Olalla@zut.edu.pl (A.S.O.); apiegat@zut.edu.pl (A.P.); psobolewski@zut.edu.pl (P.S.); 2 Instituto de Ciencia y Tecnología de Polímeros, ICTP-CSIC, Calle Juan de la Cierva 3, 28006 Madrid, Spain; 3 Department of Chemical and Biomolecular Engineering, The University of Akron, Akron, OH 44325, USA; atm34@zips.uakron.edu (A.M.); jpuskas@uakron.edu (J.P.)

**Keywords:** polyester copolymers, enzymatic degradation, nanofibers, electrospinning, fatty acid, poly(butylene succinate) (PBS)

## Abstract

Biodegradable polymers are an active area of investigation, particularly ones that can be produced from sustainable, biobased monomers, such as copolymers of poly(butylene succinate) (PBS). In this study, we examine the enzymatic degradation of poly(butylene succinate-dilinoleic succinate) (PBS-DLS) copolymers obtained by “green” enzymatic synthesis using lipase B from *Candida antarctica* (CALB). The copolymers differed in their hard to soft segments ratio, from 70:30 to 50:50 wt %. Enzymatic degradation was carried out on electrospun membranes (scaffolds) and compression-moulded films using lipase from *Pseudomomas cepacia*. Poly(ε-caprolactone) (PCL) was used as a reference aliphatic polyester. The degradation process was monitored gravimetrically via water uptake and mass loss. After 24 days, approx. 40% mass loss was observed for fibrous materials prepared from the PBS-DLS 70:30 copolymer, as compared to approx. 10% mass loss for PBS-DLS 50:50. Infrared spectroscopy (FTIR) and size exclusion chromatography (SEC) analysis were used to examine changes in chemical structure. Differential scanning calorimetry (DSC) and scanning light microscopy (LSM) revealed changes in degree of crystallinity, and changes in surface morphology, consistent with a surface erosion mechanism. We conclude that the obtained copolymers are suitable for tissue engineering applications thanks to tuneable degradation and lack of acidification during breakdown.

## 1. Introduction

Biodegradable polymers are being constantly investigated for medical applications [1], especially for tissue engineering (TE). Such polymers need to meet many important requirements to be successfully applied in TE. These include having suitable chemical properties (non-toxicity, biodegradability, and ease of sterilization), physical properties (nanofibrous, elastic, and complaint), and biological properties (biocompatibility and biofunctionality) [2]. Currently, attention is primarily focused on a large group of aliphatic polyesters, including poly(lactic acid) (PLA), poly(glycolic acid) (PGA), poly(**ε**-caprolactone) (PCL) [3], and polyhydroxyalkanoates (PHA) [4], such as poly(hydroxybutyrate) (PHB). Overall, these biobased polymers see wide use in tissue engineering due to their biodegradability, good biocompatibility, bioresorbability, and suitable mechanical properties.

However, since the 1990’s, poly(butylene succinate) (PBS) and its copolymers have gained (renewed) prominence within this aliphatic polyester family, particularly for biomedical applications [5,6,7]. PBS offers good mechanical properties and biocompatibility, as well as better processability, easier obtainability, and higher elongation at break than other aliphatic polyesters. In particular, its high ductility suggests that PBS might be a good candidate for soft tissue repair. In terms of degradation, much like the other aliphatic polyesters, PBS is susceptible to hydrolysis and, in the environment, degradation of PBS by enzymes and microorganisms yields harmless products (carbon dioxide and water). However, (but again similarly to PLA, PGA, PCL and PHB) the crystallinity of PBS plays a major role in the degradation process [8] and may limit its suitability for soft-tissue repair and tissue engineering, due to its relatively slow degradation and resorption rate. In these applications, the polymer should combine an appropriate mechanical performance and degradation profile; maintaining strength until the formation of new tissue can occur to replace the degraded material.

It is well known that polyester properties are influenced by many variables, some of which are molar mass, chemical structure, stereochemistry, hydrophilic/hydrophobic balance or chain mobility [9,10]. Consequently, copolymerization [11,12,13,14], as well as nanoadditives [15], are well-established strategies for tailoring the properties of PBS to suit various biomedical applications. Previously, we demonstrated that copolymerization of PBS with an increasing amount of dilinoleic acid (a dimerized fatty acid derivative) yielded lower crystallinity, higher susceptibility to degradation, and increased elasticity [16]. These fatty acid derivatives are obtained from vegetable oils and, thus, this new family of PBS copolymers could be entirely biobased, because PBS monomers (diol, diacid/diester) are readily available from refined biomass and biobased PBS (as well as various copolymers) is already commercially accessible [17].

Sustainability and “green chemistry” [18] can be further embraced in polyester synthesis by employing enzymatic catalysts [19], which offer much greater selectivity. This approach offers several additional advantages compared to traditional melt polycondensation [20], such as mild conditions reactions (low temperature and pressure), and no toxic organometallic initiators—particularly relevant for biomedical applications. Lipases, in particular, have been demonstrated to be powerful biocatalysts for the synthesis of polyesters when the proper conditions are used [21,22,23]. In previous work performed in our laboratory using immobilized lipase B from *Candida antarctica* (CALB) as a catalyst, we obtained PBS copolymers containing dimer linoleic diol (poly(butylene succinate-*co*-dilinoleic succinate)), (PBS-DLS) with well-defined structures and high reproducibility of the initial monomer feed ratio [24].

Our primary goal has been to obtain unique PBS-DLS copolymers, able to form flexible and soft materials for application in scaffolds for soft tissue engineering. The introduction to fatty acid moieties (dilinoleic diol), as well as using enzymatic catalysis, can be expected to significantly influence the degradation profile of these copolymers. In this work, we examine the enzymatic degradation of PBS-DLS copolymers differing in the amount of dilinoleic diol building up the soft segments (30 and 50 wt %, respectively). In the body, a range of hydrolase enzymes, present in fluids and tissues or as part of the inflammatory response, can be involved in increasing the in vivo degradation rate, as compared to hydrolysis alone [25]. It is impossible to adequately mimic these complex (and dynamic) conditions in vitro, thus it is common and useful to use model enzyme systems to gain insight in to the degradation behavior of new materials and plan future in vivo studies. Lipase, a hydrolase present in human serum and macrophages, is a common model enzyme system, as it is well-known to hydrolyze ester bonds of aliphatic polyesters in aqueous media [25] (in addition to the previously mentioned catalytic activity towards ester formation). In the present study, we utilized lipase from *Pseudomonas cepacia,* one of the most commonly used lipases for in vitro enzymatic degradation, thanks to sufficient stability and activity to yield reproducible studies [11]. The degradation process was monitored by weight loss, water uptake, and changes in medium pH as a function of degradation time, as well as Fourier Transform Infrared Spectroscopy (FTIR) and Laser Scanning Microscopy. Additionally, Differential Scanning Calorimetry (DSC) and Size Exclusion Chromatography analysis were performed to compare samples before and after degradation.

## 2. Materials and Methods

### 2.1. Materials

**Figure 1:**
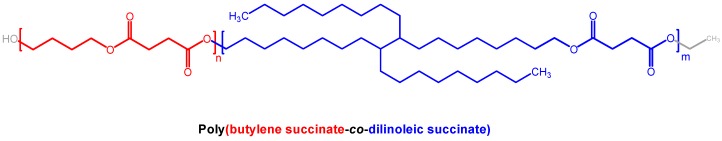
Chemical structure of synthesized PBS-DLS copolymers showing the composition of hard segments (red) and soft segments (blue).

Two aliphatic PBS copolyesters containing dimer linoleic diol moieties (PBS-DLS), differing in hard to soft segments ratios (70:30 and 50:50 wt %), were synthesized using immobilized CALB (Fermase CALB™10000, purchased from Fermenta Biotech Ltd. Mumbai and kindly provided by Enzyme Catalyzed Polymers LLC, Akron, OH, USA) as a catalyst. The synthesis was carried out using a two stage method and worked-up accordingly to the procedure described previously [24] to obtain samples with a white powder appearance after 96 h. Briefly, 1,4-butanediol (BD, ≥99%, Sigma-Aldrich, Poznań, Poland), dimer linoleic diol (DLA-OH, dimer alcohol ≥96.5%, Pripol^TM^ 2033, Croda, Gouda, The Netherlands), and diethyl succinate (DS, ≥99%, Sigma-Aldrich, Poznań, Poland) were dissolved in diphenyl ether (DE, ≥99%, Sigma-Aldrich, Poznań, Poland) (in appropriate ratios to obtain desired hard to soft segment ratio of the copolymer) with the addition of CALB as the catalyst. The mixture was reacted at atmospheric pressure at 80 °C under N_2_. The temperature was slowly increased to 95 °C and after 2 h (half of the theoretical amount of ethanol was collected) the pressure was reduced to 600 Torr. After 18 h the pressure was gradually reduced to 2 Torr and maintained for 72 h (96 h of total reaction time). After this time, the cooled products were dissolved in chloroform and filtered to remove the CALB. Products were concentrated using a rotary evaporator and precipitated by drop-wise addition into cold methanol (MeOH: ≥99.8%, POCh, Gliwice, Poland), under continuous stirring. Finally, the polymers were filtered, washed well with cold methanol, and dried in a vacuum oven at 40 °C. The mass feed of all monomers was varied in order to obtain two materials with butylene-succinate (BS) units as hard segments and succinate-dimer linoleic diol units (DLS) as soft segments in approximately 50:50 and 70:30 wt % ratios. The obtained copolymers with hard to soft segments ratios of 70:30 and 50:50 wt % will be abbreviated in this work as PBS-DLS 70:30 and PBS-DLS 50:50, respectively. The structural formula of the copolymers comprising hard and soft segments respectively is illustrated in Figure 1.

### 2.2. Characterization of PBS-DLS Enzymatically Obtained Copolymers

The chemical structures of purified PBS-DLS 70:30 and 50:50 copolymers were verified by nuclear magnetic resonance (^1^H NMR, ^13^C NMR) and Fourier transform infrared spectroscopy (FTIR). The ^1^H NMR (128 scans, 1 s relaxation delay) and ^13^C NMR (5120 scans, 1 s relaxation delay) spectra were recorded in chloroform-d (CDCl_3_: 99.8% atom D) on a TM Bruker DPX 400 (400 MHz) spectrometer (Bruker, Germany) with tetramethylsilane (TMS) as internal reference. FTIR transmission spectra (4000 to 400 cm^−1^, 2 cm^−1^ resolution, 32 scans), were obtained on a Bruker ALPHA spectrometer with an Attenuated Total Reflectance (ATR) cell. Intrinsic viscosity [*η*] measurements were performed using an Ubbelohde capillary viscometer (Labit, Koczargi Nowe, Poland) (*K* = 0.00342) immersed in a water bath at 25 °C. Samples were dissolved in chloroform (0.125 g/25 cm^3^) and results were calculated using the Solomon–Ciuta Equations (1)–(3) [26].

(1) $$\;\eta =\frac{\sqrt{2(\eta_{w}-\ln\eta_{r})}}{c}$$

(2) $$\;\eta_{w}=\eta_{r}-\;1$$

(3) $$\;\eta_{r}=\frac{t_{1}}{t_{o}}$$

where:

*t_o_*—average flow time for solvent [s]

*t*_1_—average flow time for polymer solution [s]

*c*—concentration of polymer solution [g/100 mL]

Qualitative size exclusion chromatography (SEC) measurements were performed in order to better understand and confirm the degradation results, in a set-up consisting of an Agilent 1260 Infinity Isocratic Pump (Agilent Technologies, Santa Clara, CA, USA), a Wyatt OPTILAB T-rEX interferometric refractometer (Wyatt Technology, Santa Barbara, CA, USA), a Wyatt DAWN HELOS-II multi-angle static light scattering detector (MALS) (Wyatt Technology, Santa Barbara, CA, USA) with a built-in dynamic light scattering (DLS) module, an Agilent 1260 Infinity Standard Autosampler (Agilent Technologies, Santa Clara, CA, USA) and 6 StyragelVR columns (HR6, HR5, HR4, HR3, HR1, and H0.5). The columns were thermostatted at 35 °C and tetrahydrofuran (THF), continuously distilled from CaH_2_, was used as the mobile phase at a flow rate of 1 mLmin^−1^. Previous to the analysis, samples were dissolved in THF (the same employed as mobile phase in the instrument) at 2–3 mg·mL^−1^ and filtered. The dRI signal was analyzed qualitatively.

### 2.3. Sample Preparation for Degradation Studies

Commercial PCL was used as a reference and positive control (CAPA 6430, Perstorp, M_n_ = 43,000 g/mol), as it has well-established biomedical applications [3], while PBS homopolymer is very resistant to enzymatic degradation [10,27] due to combination of high crystallinity and a high melting temperature. The PCL and enzymatically synthesized PBS-DLS 70:30 and 50:50 were processed by both hot-press melting and electrospinning, in order to assess the degradation profiles of the materials in the shape of films and fibrous membranes (scaffolds). Thin polymer films (100 µm) were prepared in a Remi-Plast hot-pressing machine (Remi-Plast, Czerwonak, Poland) at temperature of 120 °C. Electrospun mats of similar thickness (~100–150 µm) were prepared by means of a standard horizontal electrospinning setup equipped with a ASCOR MED AP14 (Ascor Med, Warsaw, Poland) pump, a Gamma High Voltage Research (Gamma High Voltage, Ormond Beach, FL, USA) voltage source connected to a 21 gauge needle, and a grounded flat collector covered with aluminum foil. PCL polymeric solution was prepared by dissolving 20 wt % of the material in a DMF:DCM 5:5 *v*/*v* solvent mixture. PBS-DLS 70:30 and PBS-DLS 50:50 solutions were prepared by dissolving 25 and 35 wt % respectively in a CHCl_3_:MeOH 7:3 *v*/*v* solvent mixture. All solutions were stirred at room temperature overnight and polymers were pre-dried in a vacuum oven before their preparation. The electrospinning process parameters are summarized in Table 1. The concentrations and parameters used are optimal for obtaining fibers free of defects or drops. The obtained mats were dried under vacuum for 48 h in order to eliminate any residual solvents. Samples in the shape of discs (6 mm diameter) were punched-out from the polymeric film and mats to perform the degradation studies.

### 2.4. Enzymatic Degradation

Enzymatic degradation was carried out inside a laminar flow cabinet [28]. The degradation media consisted of a solution of lipase enzyme (EC3.1.1.3, from *Pseudomonas cepacia* (Sigma Aldrich, Poznań, Poland) (25 units/mL) prepared in Dulbecco’s PBS buffer (Sigma Aldrich, Poznań, Poland) (pH range 7.1–7.2), with sodium azide added to avoid bacterial contamination. Each sample, in the form of a 6 mm diameter disc, was placed in the well of a 48-well plate and covered with 1.5 mL of degradation media. Plates were then incubated at 37 °C on a rocker (60 rpm) for 24 days and samples were collected for analysis at 24 h, 48 h and every 96 h thereafter. In order to maintain enzymatic activity, the degradation medium was changed every 48 h. At the specific time points, samples were removed, washed with distilled water, and weighed (*n* = 3).

### 2.5. Characterization of Processed Materials before and after Degradation

Water uptake and weight loss were determined gravimetrically using an analytical balance with an accuracy of 0.01 mg. At the specific time points, samples were removed from the media, washed thoroughly with distilled water, and weighed wet. Then discs dried at 40 °C under a pressure of ~30 mmHg for 10 days and weighed dry. The initial mass, before degradation, was indicated as *m*_0_, while the wet mass, immediately after degradation was indicated as *m*_w_ (wet), and the mass after drying as *m*_d_.

The water uptake (*P*) was then calculated from equation:

(4) $$\;P=\frac{m_{w}-m_{d}}{m_{d}}\cdot 100\%$$

The weight loss (*D*) was calculated from equation:

(5) $$\;D=\frac{m_{0}-m_{d}}{m_{0}}\cdot 100\%$$

Changes in the chemical structure of films and fiber membranes (scaffolds) before and after degradation were evaluated by ATR-FTIR spectroscopy (measured under previously mentioned conditions).

TA Instruments Q1000 (TA Instruments, New Castle, DE, USA). Approximately 3 mg of sample (sealed in aluminum pans) was subjected to a triple heating-cooling-heating cycle −90 to 190 °C at a rate of 10 °C/min under N_2_ atmosphere. Glass transition temperature (*T*_g_), melting temperature (*T*_m_) and crystallinity were evaluated from the heating run and before/after degradation. In all the cases, glass transition temperature (*T*_g_) was calculated as the midpoint of the transition. The crystalline phase content in the hard segments (*X_c,h_*%) for PBS-DLS 70:30 and 50:50 was calculated following the Equations (6) and (7), while the total content of crystalline phase in PCL (*X_c,tot_*%), was calculated from Equation (8):

*X_c,h_*% = (0.7 × Δ*H_m_*/Δ*H*^0^*_m_*) × 100 (6)

*X_c,h_*% = (0.5 × Δ*H_m_*/Δ*H*^0^*_m_*) × 100 (7)

*X_c,tot_*% = Δ*H_m_*/Δ*H*^0^*_m_* × 100 (8)

where Δ*H*^0^*_m_* is Δ*H_m_* =110.3 J/g^−1^ for 100% crystalline PBS [29] and Δ*H_m_* =135.3 J/g^−1^ for 100% crystalline PCL [30].

Changes in the surface morphology (roughness, cracks, pores) were examined by Laser Scanning Microscopy (LSM) using a Keyence VK9700 LSM equipped with a 408 nm violet laser (Keyence, Itasca, IL, USA). Images were collected at maximum resolution (2048 × 1536) with a 10% and 30% intensity setting of the laser for the 20× (0.3 NA) and 50× (0.95 NA) objectives respectively.

## 3. Results

### 3.1. Chemical Structure Analysis

**Figure 2:**
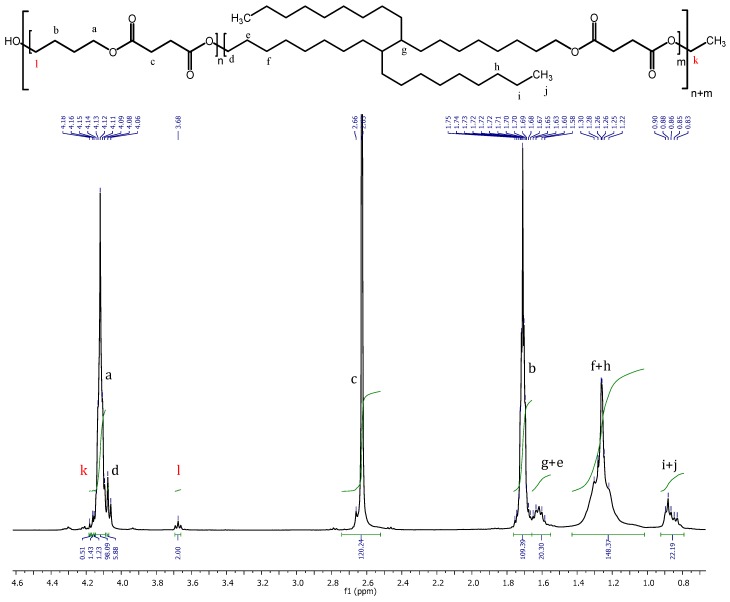
^1^H NMR of poly(butylene succinate-*co*-dilinoleic succinate) 70:30 copolyester.

^1^H NMR and ^13^C NMR results confirmed the successful synthesis of the CALB catalyzed PBS:DLS copolymers. Figure 2 presents the ^1^H NMR spectrum of PBS-DLS 70:30 copolymer.

In ^1^H NMR, signals appearing at: *δ*^1^H = 4.12 ppm are due to four protons from BD adjacent to the oxygen (a), *δ*^1^H = 2.63 ppm are signals from four protons from DS (c), *δ*^1^H = 1.72 ppm are from BD (b). Following signal are characteristic for soft segments DLS units: *δ*^1^H = 1.62 ppm (20H, from DLA-OH, g + e), *δ*^1^H = 1.25 (52H, from DLA-OH, f + h), 0.85 (10H, from DLA-OH, i + j). Low intensity resonance ascribed to the BD end-groups appears at 3.68 ppm (2H, **l**, normalized to value 2.00) (^13^C NMR confirmed the absence of DLA-OH end groups. Figures S2 and S3). Diethyl succinate end-groups at 4.16 ppm and at 1.25 are partially/total overlapped with BD and DLA-OH signals respectively.

As detailed in the Supplementary Materials, analysis of the ^1^H NMR spectra showed that the final achieved copolymer compositions were in a good agreement with the initial monomer feed (BD:DS:DLA-OH molar feed/compositions; PBS-DLS 70:30 initial 45:50:5, final 45:50:5; PBS-DLS 50:50 initial 50:39:11, final 50:41:9) (Supplementary Materials Figures S1 and S2; Supplementary Materials Tables S1 and S2) [24]. In addition, the number-average molecular weight (M_n_) calculated from ^1^H NMR (Supplementary Materials) resulted in an M_n_ = 11,590 g/mol for PBS-DLS 70:30 and M_n_ = 27,440 g/mol for PBS-DLS 50:50. The higher value obtained for PBS-DLS 50:50 is on account of the higher amount of DLA-OH present in the polymer backbone. An analogous relationship was also observed for the intrinsic viscosity measurements, where for PBS-DLS 70:30 η = 0.40 dL/g and for 50:50 copolymer η = 0.56 dL/g.

Taking into account the related literature and our previous experience with enzymatically synthesized PBS and PBS copolymers, lowering of the vacuum in a stepwise manner and the usage of high boiling point and high log *P* (octanol/water partition coefficient of diphenyl ether 4.21) solvent, helps the formation of a high molecular weight materials avoiding premature deactivation of the catalyst and precipitation of the system for medium conversions [24,31,32,33]. Thus, the synthesis conditions chosen helped to retain the initial desired ratio of hard to soft segments and favor the formation of high molecular weight PBS-DLS materials that ensured their further processing into fibers and films.

### 3.2. Enzymatic Degradation of Film and Fibrous Materials

**Figure 3:**
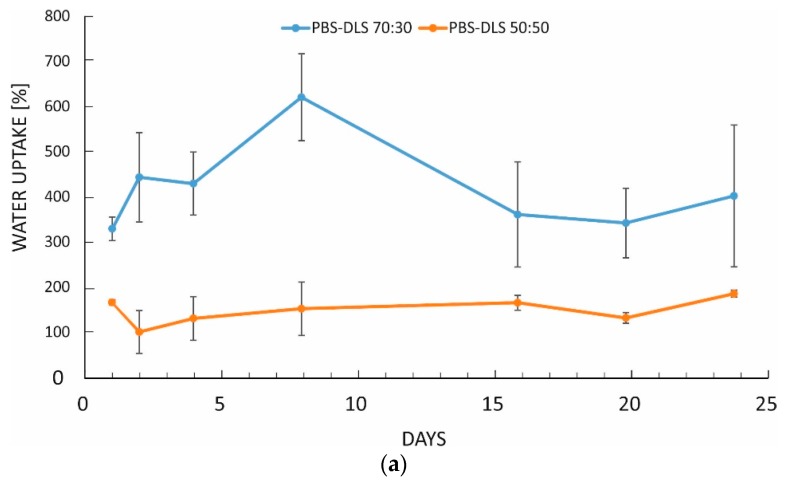

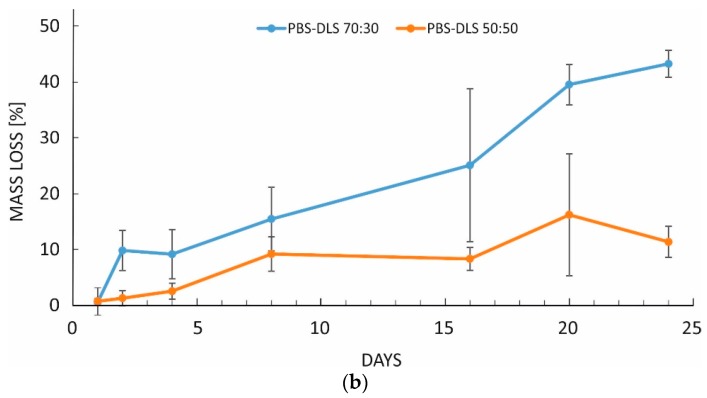
Water uptake (**a**) and mass loss (**b**) for electrospun fiber mats of PBS-DLS 70:30 and PBS-DLS 50:50 (mean ± SD, *n* = 3).

The progress of enzymatic degradation was monitored for film and fibrous materials differing in segmental composition. The surface area available for degradation plays an important role, as in contrast to hydrolytic degradation, which for PBS copolymers is typically a bulk process, enzymatic degradation can be expected to occur via surface erosion [5]. Here, the mass loss and water uptake values for bulk materials (films, ~100 µm) were low and almost constant over the observation period of 24 days: Mass loss was at the same level of 3.2% for both PBS-DLS 70:30 and PBS-DLS 50:50 copolymers, while water uptake was higher for PBS-DLS 70:30 (8.7%) than for PBS-DLS 50:50 (5.4%). The difference in water uptake, while small, can be explained by the higher content of hydrophobic fatty acid sequences in PBS-DLS 50:50 and higher M_n_ as compared to PBS-DLS 70:30. In the case of electrospun mats, the fibrous morphology had a very pronounced effect on water uptake and mass loss—dramatically increasing both, as compared to bulk materials—as can be seen from Figure 3a. For the PBS-DLS 70:30 copolymer, containing a higher amount of short butylene succinate hard segments, it can be clearly seen, that the much greater surface area of fibrous materials along with the segmental composition has resulted in a much higher water uptake, ranging from 400% to 600%, as compared to approx. 100% for PBS-DLS 50:50, which contains more long chain hydrophobic fatty acid sequences. As can be seen from Figure 3b, fibrous mats show increasing mass loss over time: after 24 days, mass loss exceeds 40% for PBS-DLS 70:30 and is approx. 10% for PBS-DLS 50:50. The much more rapid degradation of fibrous mats, as compared to films, is likely due to the much greater effective surface area, in agreement with a surface erosion mechanism.

**Figure 4:**
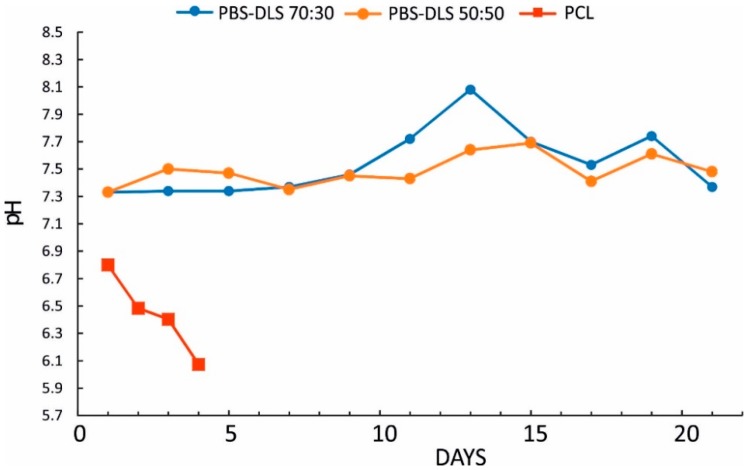
Changes in the pH of degradation media during degradation for PBS-DLS 70:30, PBS DLS 50:50, and PCL films.

**Figure 5:**
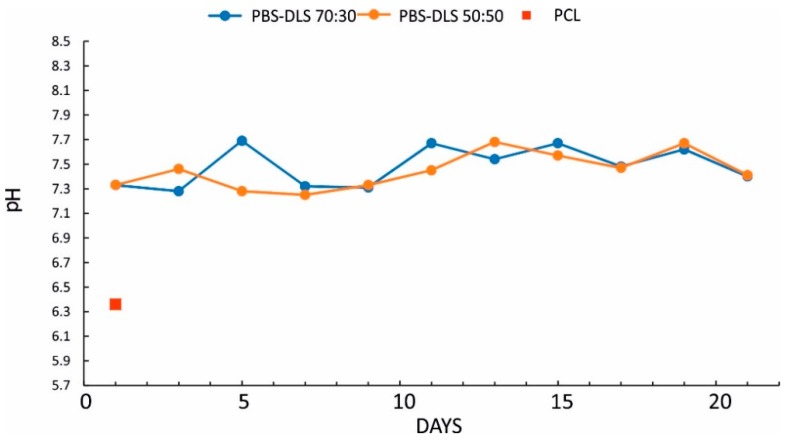
The changes in the pH of degradation media during degradation for PBS-DLS 70:30, PBS-DLS 50:50, and PCL electrospun fiber mats.

Importantly, no acidification of the degradation media was observed during the degradation process of films (Figure 4) and electrospun mats (Figure 5), for either copolymer composition. The reference material and positive control PCL exhibits very fast degradation, along with a sharp drop in pH (as low as pH = 6). Again, the degradation proceeds much faster for electrospun PCL mats; these materials completely disintegrate after 24 h.

**Figure 6:**
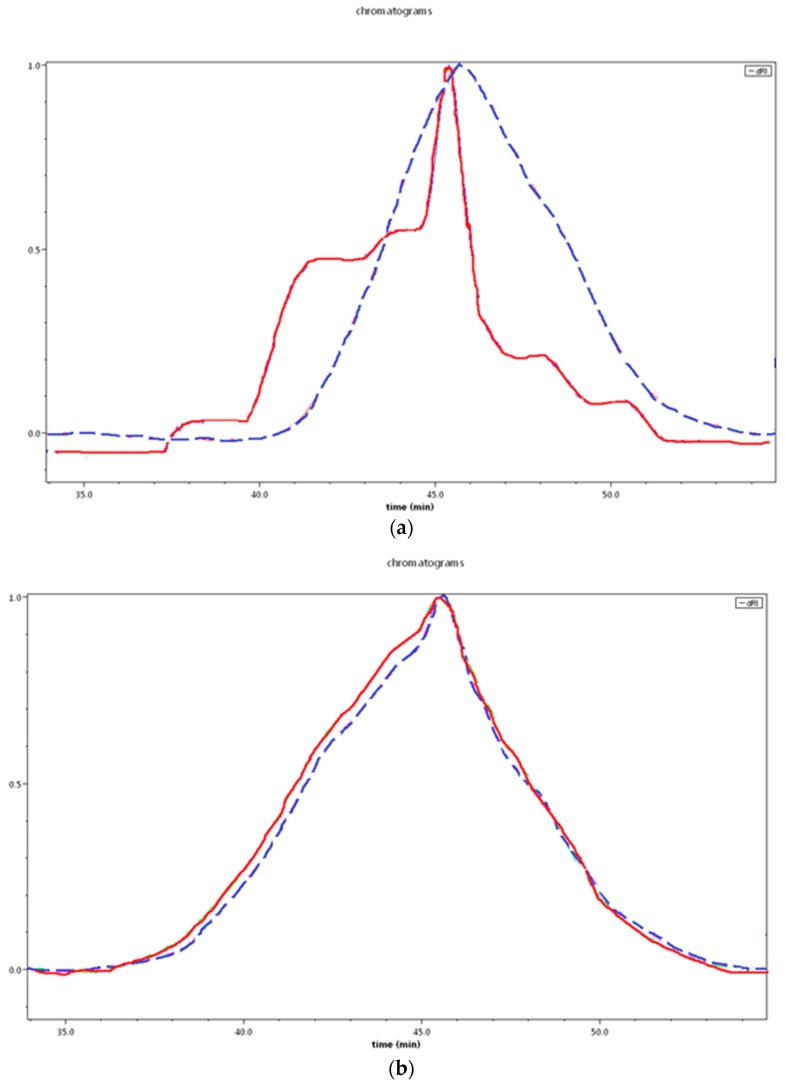
High resolution SEC chromatograms of films (**a**) PBS-DLS 70-30 and (**b**) PBS-DLS 50:50, before (solid line, red) and after (dashed line, blue) degradation (24 days).

In order to reveal changes in M_n_ of degraded samples, SEC was carried out and the dRI signal was analyzed qualitatively. Figure 6 shows the traces before and after degradation for films materials. The trace of PBS-DLS 70:30 before degradation is multimodal, clearly showing the populations with various molecular weights, typical of polycondensation reactions. After degradation (24 days), no differences are observed for PBS-DLS 50:50 (Figure 6b), while PBS-DLS 70:30 (Figure 6a) showed some molecular weight breakdown, especially from the higher molecular weight populations. These results are consistent with a surface erosion mechanism, as one would expect small molecular weight breakdown products to be dissolved in the media.

**Figure 7:**
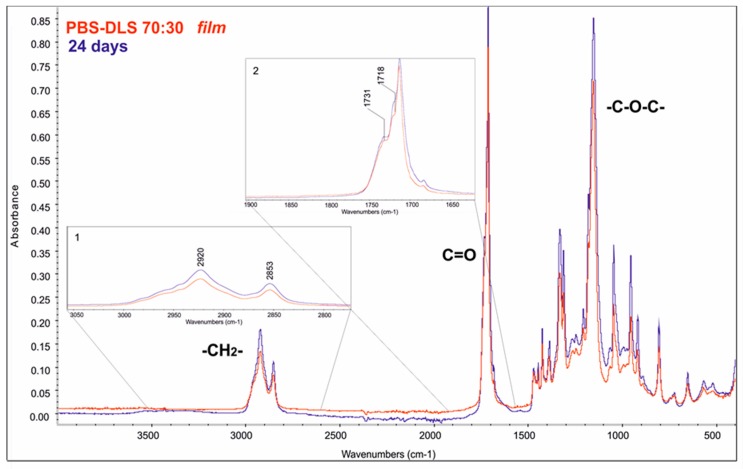
FTIR-ATR spectra of PBS DLS 70:30 films, before and after the enzymatic degradation. Inset (1): 2920, 2853 cm^−1^ correspond to aliphatic soft segments backbone. Inset (2): peaks corresponding to ester carbonyl backbone: 1718 and 1731 cm^−1.^

The chemical structure of materials was monitored with infrared spectroscopy (ATR–FTIR) (here we show only PBS-DLS 70:30, Figure 7). Three characteristic regions of both PBS-DLS 70:30 and PBS-DLS 50:50 copolyesters are highlighted: (1) the two peaks at 2920 and 2853 cm^−1^ corresponding to absorption by methylene –CH_2_– stretching groups of the aliphatic soft segments, (2) the peak at 1718 cm^−1^ corresponding to absorption by carbonyl C=O bond stretching, and (3) the peak at 1153 cm^−1^ corresponding to the ester C–O–C bond stretching. As can be seen from Figure 7 (PBS-DLS 70:30, bulk material (film) in region 1, at 2920 and 2853 cm^−1^, there is only a slight increase in absorbance after 24 days of degradation, which is consistent with the SEC observation where we do not see changes in M_n_ and confirms that the polymer is degrading via surface erosion.

**Figure 8:**
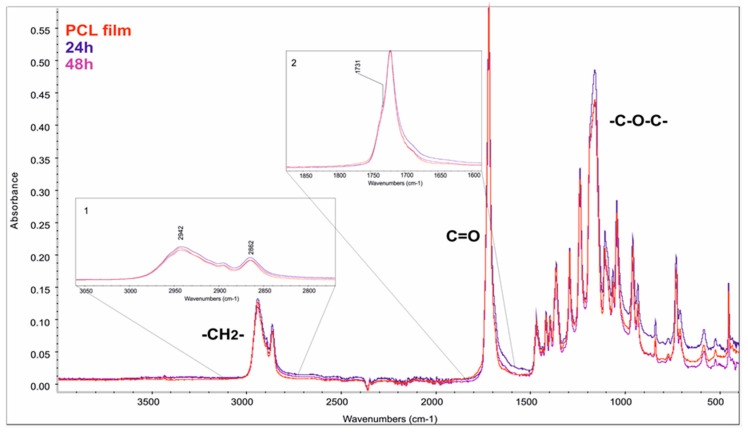
FTIR-ATR spectra of PCL films, before and after the enzymatic degradation, Inset (1) show changes in peaks at 2942 and 2862 cm^−1^ ascribed to aliphatic chains. Insets 2: Peak corresponding to C=O bonds of ester groups.

Figure 8 shows changes in IR spectra for films prepared from PCL homopolymer. As can be seen from this figure in region 1, two peaks, 2942, 2862cm^−1^, indicate the absorbance by stretching of methylene –CH_2_– groups of aliphatic soft segments. The peak in region 2, at 1731 cm^−1^, and the peak at 1159 cm^−1^, corresponds to C=O and C–O–C bonds, respectively, confirming the presence of carbonyl bonds of ester groups. Due to rapid degradation of the material, it was only possible to obtain spectra after 24 and 48 h. For PCL films, the absorbance in region 1, at 2942 and 2862 cm^−1^, increases following degradation. In region 2, with the peak at 1731 cm^−1^, there is a shift of the spectrum to the lower values and band at 1159 cm^−1^, showing an increase in absorbance as observed after 24 h of degradation.

### 3.3. Thermal Properties and Surface Morphology of Films and Fiber Materials

**Figure 9:**
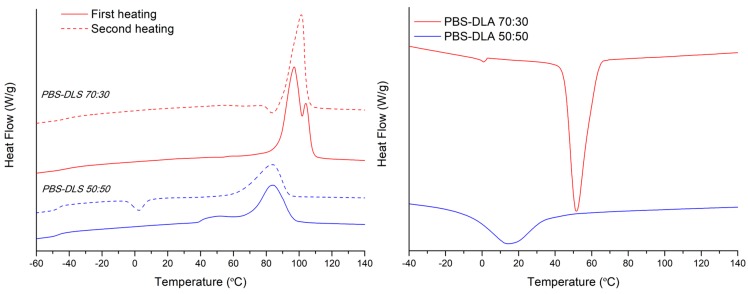
DSC thermograms. **Left**: first (solid line) and second heating (dashed line) scans of polymer powders (after synthesis) of PBS-DLS 50:50 (blue) and PBS-DLS 70:30 (red) copolymers. **Right**: cooling scan of polymer powders (after synthesis) PBS-DLS 50:50 (blue) and PBS-DLS 70:30 (red) copolymers.

Table 2 summarizes the thermal properties of the synthesized copolymers and Figure 9 shows their DSC traces. As can be seen, both copolymers are semicrystalline as the thermal behavior is characterized by the presence of a glass transition at around −40 °C and an endothermic peak at higher temperatures related with the melting of crystalline part. However, compared to the values of PBS homopolymer (*T*_m_ = 113 °C) [34], the increase in DLA–OH content leads to a melting point decreasing in temperature value and lower in enthalpy (Δ*H_m_*). Further, PBS-DLS 50:50 has a broad melting peak and reaches the lowest value of melting enthalpy, probably due to a decrease in the perfection of the crystalline phase. In addition, the glass transition step height is more evident for this system due to the higher amount of amorphous part (soft segments). Although PBS homopolymer, as well as both copolymers, have the capability to crystallize before melting (cold-crystallization), the copolymerization with DLA–OH affects the area under the exothermic peak, (Δ*H_cc_*) lowering the ability of the butylene succinate sequences to crystallize. In addition, the DSC curve for PBS-DLS 50:50 shows a reduction in the cold crystallization temperature at 3 °C, suggesting that the highest amount of DLA-OH increased the chain mobility of the system [35]. Finally, from the cooling run, it can be seen that the *T*_c_ behaves similarly to the *T*_m_, decreasing as a function of the DLA–OH content. Regarding the *T*_g_ values, the DSC results showed just one *T*_g_ value for the PBS-DLS 70:30 and PBS-DLS 50:50 copolymers (Table 2), with both having an intermediate value between *T*_g_ of PBS homopolymer (~−43 °C) [27] and neat DLA (~55 °C) [36]. Due to this, and taking into account the synthesis procedure, the most likely segmental distribution for the obtained copolymers is a random distribution. 

**Figure 10:**
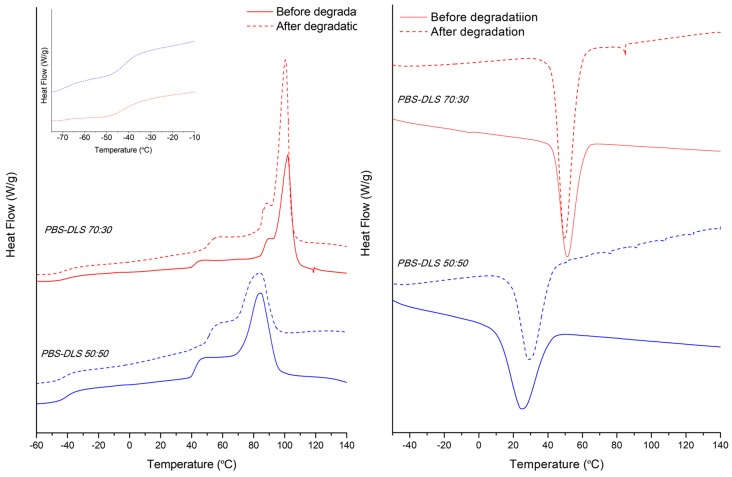
**Left**: First heating scan of films before (solid line) and after (dashed line) degradation with a *T*_g_ region detail of degraded samples. **Right**: Cooling scan of films before (solid line) and after (dashed line) degradation. PBS-DLS 50:50 (blue) and PBS-DLS 70:30 (red) copolymers.

**Figure 11:**
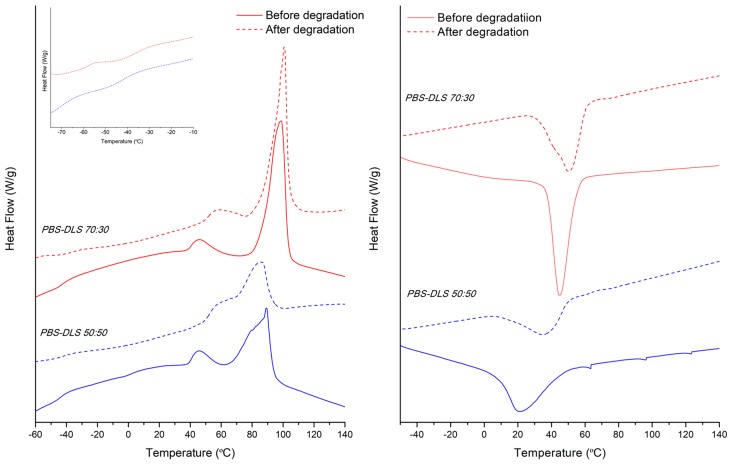
**Left**: First heating scan of fibers before (solid line) and after (dashed line) degradation with a *T*_g_ region detail of degraded samples. **Right**: Cooling scan of fibers before (solid line) and after (dashed line) degradation. PBS-DLS 50:50 (blue) and PBS-DLS 70:30 (red) copolymers.

With respect to the processed materials, films and fibers, before and after degradation, the thermal behavior was analyzed from the first heating. The characteristic transition temperatures and DSC traces are presented in Table 2 and Figure 10 (films) and Figure 11 (fibers). The processing parameters (hot melt pressing versus electrospinning from solution) did not strongly affect the melting temperature, *T*_m_. However, the processing of materials did have an effect on the crystallization temperature *T*_c_, which was found to be higher for PBS-DLS 70:30 film, as compared to the fibrous mat fabricated from this copolymer (Table 3). On the other hand, for PBS DLS 50:50, with a lower content of crystallizable hard segments, a higher *T*_c_ was observed for fibrous and film materials, as compared to the as-synthesized copolymer (powder). Regarding the crystallinity, it appears that during the electrospinning process the molecules of PBS-DLS had less time to crystallize, compared to the film forming process, which allows for the crystallization of the PBS, as can be seen from the calculated *Χ_c,h_* values and the DSC cooling process of the powder materials (Figure 9) that can simulate the cooling during the hot-press film forming [35]. 

Meanwhile, as can also be seen from DSC thermograms (Figure 10 and Figure 11), the degradation process has an effect on melting enthalpy, Δ*H_m_*. In general, for processed PBS-DLS 70:30 fibers and films after degradation, there is an increase in the melting enthalpy, as a direct consequence of the degradation affecting mainly the amorphous part. The same trend occurs for the PBS-DLS 50:50 fibers; however, after degradation the films retain the same Δ*H_m_* as before, which is in agreement with the overall results (mass loss, LSM and SEC) indicating slower degradation for this material. Interestingly, as can be seen from the inset in Figure 11, the degradation process has a greater effect on the *T*_g_ of the fibers, compared to the as-synthesized powder. In particular, for the PBS-DLS 70:30 fibers, there is a clear split into 2 different *T*_g_, one at −35 °C, closer to that of PBS homopolymer, and the other at −58 °C, closer to that of neat DLA. This is direct evidence of the hydrolysis of the linkage between copolymer hard and soft segments contributing to the highest degradation rate observed for this material. This phenomenon appears to start to occur in the PBS-DLS 50:50 fibers after degradation, but is not as evident as in the PBS-DLS 70:30.

**Figure 12:**
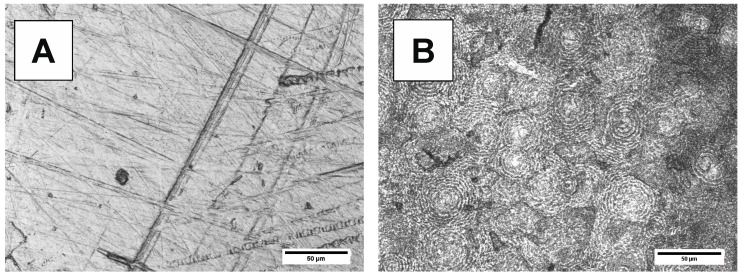
LSM micrographs of PBS-DLS 70:30 films (**A**) before and (**B**) after 24 days of degradation.

**Figure 13:**
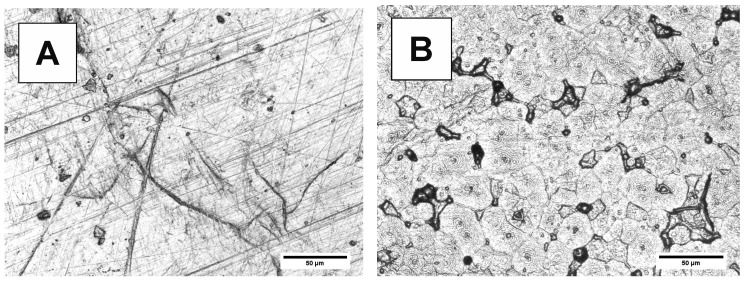
LSM micrographs of PBS-DLS 50:50 films (**A**) before and (**B**) after 24 days of degradation.

**Figure 14:**
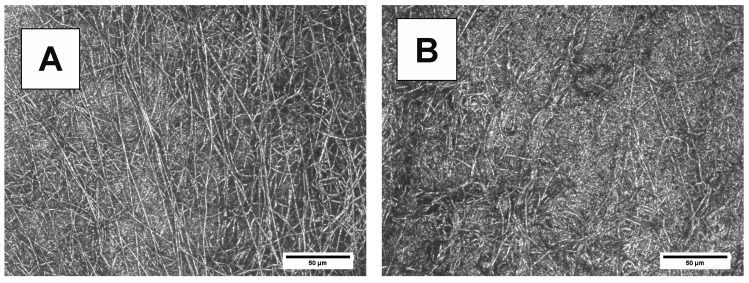
LSM micrographs of PBS DLS 70:30 electrospun fibers (**A**) before and (**B**) after 24 days of degradation.

**Figure 15:**
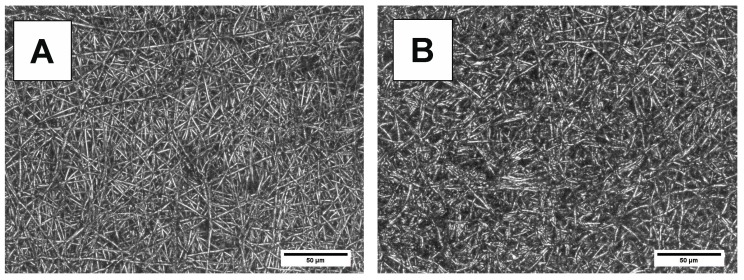
LSM micrographs of PBS-DLS 50:50 electrospun fibers (**A**) before and (**B**) after 24 days of degradation.

Changes in the surface morphology of samples before and after degradation are shown in Figure 12, Figure 13, Figure 14 and Figure 15. Polymeric films were prepared by melting in hot press, followed by cooling. The LSM micrographs of both bulk materials (Figure 12A and Figure 13A) before degradation show surfaces with cracks, reflecting the Teflon foil surface used during hot pressing. After degradation (Figure 12B and Figure 13B), the PBS-DLS 70:30 copolyester exhibits banded morphology, attributed to the spherulites formed during the crystallization. However, the typical periodic distance along the radial direction is not observed. It is possible that the amorphous region of the spherulites degraded, while the crystalline component still remains after the degradation. The surface morphology of the PBS-DLS 50:50 copolyester does not show as pronounced spherulitic forms (Figure 13B). Further, this copolyester shows holes due to the degradation and many un-banded structures. This surface morphology can be related to higher content of hydrophobic fatty acid soft segments, which may be resistant to enzymatic lysis. The LSM micrographs are consistent with the DSC analysis, which indicated lower crystallinity of PBS-DLS 50:50 copolyester.

The morphology of electrospun mats is presented in Figure 14 and Figure 15. Following degradation, micrographs of PBS-DLS 70:30 copolyester (Figure 14) reveal many regions with disrupted fibers, thus reflecting the observations from mass loss and water uptake, which indicated more pronounced degradation of these samples after 24 days. In the case of PBS-DLS 50:50 (Figure 15), more fibers are visible and fewer differences from the pre-degradation micrograph can be observed. This is consistent with the previous results and explained by the greater proportion of hydrophobic fatty acid soft segments that may segregate to the surface.

## 4. Conclusions

Our work describes the enzymatic degradation of poly(butylene succinate-*co*-dilinoleic succinate) (PBS-DLS) copolymers, which can be synthesized from sustainable, biobased monomers using enzymatic catalysis. The incorporation of dimer fatty acid soft segments yields thermoplastic elastomers with good processability, well-suited to biomedical applications. The enzymatic degradation studies presented here indicate that an increase in fatty acid soft segments is associated with lower water uptake and mass loss, indicating slower degradation. Additionally, preparing samples in the form of electrospun fiber mats had a marked influence on degradation, indicating that the effective surface area plays a pronounced role, consistent with an erosive degradation mechanism. Importantly, we do not observe any trend towards acidification of the media during the degradation process. Overall, we conclude that these copolymers are well suited to applications in biomedicine, such as tissue engineering, thanks to their attractive processability (melt compression moulding and electrospinning) and tuneable degradation behaviour, which does not generate acidic breakdown products.

## Figures and Tables

**Table 1:** Electrospinning conditions for PCL, PBS-DLS 70:30 and PBS-DLS 50:50 copolymers.

Material	Rate [mL/h]	Distance [cm]	Voltage [kV]	Solvents/Concentration (*v*/*v*, wt %)	Temp [°C]	Humidity [%]
PBS-DLS 70:30	1	15	15	CHCl_3_:DCM(7:3)/25	23	34
PBS-DLS 50:50	1	15	15	CHCl_3_:DCM(7:3)/35	23	28
PCL	3	18	18	DMF:DCM(1:1)/20	25	30

**Table 2:** Thermal behavior of PBS-DLS 70:30 and 50:50 copolymers as synthesized (powder).

Sample	*T*_c_ (J/g)	Δ*H*_c_ (J/g)	^II^*T_g_* (°C)	^II^*T*_cc_ (°C)	^II^ Δ*H_cc_* (J/g)	^II^*T*_m_ (°C)	^II^ Δ*H_m_* (J/g)	^a^*Χ_c,h_* (%)
PBS-DLS 70:30 powder	51.8	62.6	−43.3	78.8	4.4	101.3	57.3	33.6
PBS-DLS 50:50 powder	13.9	43.5	−46.4	2.9	5.8	84.2	45.1	17.8

Crystallization temperature (*T*_c_); glass transition temperature (*T*_g_); cold-crystallization temperature (*T*_cc_); melting temperature (*T*_m_); crystallization, cold-crystallization and melting enthalpies (Δ*H_c_*, Δ*H_cc_* and Δ*H_m_*); crystallinity from hard segments (*Χ_c,h_*); crystallinity total content (*Χ_c,tot_*); calculated from the DSC cooling run; ^II^ calculated from the second DSC heating run; ^a^ calculated from the second DSC heating run using Equations (6) and (7).

**Table 3:** Thermal behavior of PBS-DLS 70:30 and 50:50 copolymers before and after degradation.

Sample	Before degradation							After degradation				
	^I^*T*_g_ (°C)	^I^*T*_m_ (°C)	^I^ Δ*H_m_* (J/g)	^a^*Χ_c,h_* (%)	^b^*Χ_c,tot_* (%)	*T*_c_ (°C)	^II^*T*_g_ (°C)	^I^*T*_g_ (°C)	^I^*T*_m_ (°C)	^I^ Δ*H_m_* (J/g)	*T*_c_ (°C)	^II^*T*_g_ (°C)
PCL film	−61.2	65.0	77.8	-	57.5	24.9	−71.2	n.o.	61.4	58.7	30.1	n.o.
PBS-DLS 70:30 powder	−42.8	97.0	83.3	53.0	-	51.7	−43.3	-	-	-	-	-
PBS-DLS 70:30 fiber	−43.0	99.0	50.1	33.0	-	44.9	−43.8	−35.1 −57.9	100.8	56.8	50.2	-
PBS-DLS 70:30 film	−40.1	102.0	52.6	33.4	-	51.0	−42.4	−43.7	100.5	60.8	49.5	−43.5
PBS-DLS 50:50 powder	−47.0	84.2	61.1	27.7	-	14.0	−46.4	-	-	-	-	-
PBS-DLS 50:50 fiber	−44.1	89.3	41.6	18.8	-	21.0	−44.6	−41.6	84.7	47.7	36.0	−45.7
PBS-DLS 50:50 film	−40.6	84.8	59.6	27.0	-	24.6	−44.7	−42.2	84.0	60.4	29.2	−44.1

Glass transition temperature (*T*_g_); crystallization temperature (*T*_c_); melting temperature (*T*_m_); melting enthalpie (Δ*H_m_*); crystallinity from hard segments (*Χ_c,h_*); crystallinity total content (Χ*_c,tot_*); ^I^ calculated from the first DSC heating run; calculated from the DSC cooling run; ^II^ calculated from the second DSC heating run; ^a^ calculated from the first DSC heating run using Equations (6) and (7); ^b^ calculated from the first DSC heating run using Equation (8); n.o.-transition not observed on the thermograms.

## Supplementary Materials

The following are available online at http://www.mdpi.com/2073-4360/10/6/688/s1.
