# Supplementary material for: Enzymatic Degradation of Poly(butylene succinate) Copolyesters Synthesized with the Use of Candida antarctica Lipase B
Source: Polymers (Basel). 2018 Jun 20;10(6):688. doi: 10.3390/polym10060688 (PMC6404136; doi:10.3390/polym10060688)
Supplement: Supplementary file 1 [file polymers-10-00688-s001.pdf]

Enzymatic degradation of poly(butylene succinate) copolyesters synthesized with the use of *Candida antarctica* lipase B

Aleksandra Wcisłek[1], Agueda Sonseca Olalla[1,2], Andrew McClain[3], Agnieszka Piegat[1], Peter Sobolewski[1], Judit Puskas[3], Miroslawa El Fray[1,*]

[1] West Pomeranian University of Technology, Szczecin, Faculty of Chemical Technology and Engineering, Functional Materials and Biomaterials, Al. Piastow 45, 71-311 Szczecin, Poland
[2] Instituto de Ciencia y Tecnología de Polímeros, ICTP-CSIC, Calle Juan de la Cierva 3, 28006, Madrid, Spain
[3] Department of Chemical and Biomolecular Engineering, The University of Akron, Akron, OH, 44325, USA

Supplementary Materials

Table of contents:

- SI.1 ^{1}H NMR analysis

- SI.2 ^{13}C NMR analysis

SI.1 ¹H NMR analysis

Table SI.1. Characteristic ¹H NMR signals used for PBS-DLS 70:30 and 50:50 copolymers calculations

	Monomer	Proton	ppm	Number of H [n]	Integral [I]
	DS	c	2.6	4	120
**PBS:DLS 70:30**	BD	b	1.7	4	110
	DLA-OH	f+h	1.25	52	148
	DS	c	2.6	4	269
**PBS:DLS 50:50**	BD	b	1.7	4	217
	DLA-OH	f+h	1.25	52	648

• From Table SI.1 experimental molar % of DS, BD and DLA-OH can be calculated from equations (SI 1-3):

(SI 1) $[Molar\ \%\ DS] = \frac{\frac{I_{2.6}}{n_{2.6}}}{\frac{I_{2.6}}{n_{2.6}}+\frac{I_{1.25}}{n_{1.25}}+\frac{I_{1.7}}{n_{1.7}}}$; **(SI 2)** $[Molar\ \%\ BD] = \frac{\frac{I_{1.7}}{n_{1.7}}}{\frac{I_{2.6}}{n_{2.6}}+\frac{I_{1.25}}{n_{1.25}}+\frac{I_{1.7}}{n_{1.7}}}$; **(SI 3)** $[Molar\ \%\ DLA-OH] = \frac{\frac{I_{1.25}}{n_{1.25}}}{\frac{I_{2.6}}{n_{2.6}}+\frac{I_{1.25}}{n_{1.25}}+\frac{I_{1.7}}{n_{1.7}}}$

were Molar % DS, Molar% BD and Molar % DLA-OH are related to the experimental molar content of the succinate, butanediol and dilinoleic units on a PBS:DLS copolymer.

• In addition, M_n can be approximately calculated with equation (SI 4):

$$\textbf{(SI 4)}\ [DP_{n+m}] = \frac{\frac{I_{2.6}}{n_{2.6}}+\frac{I_{1.7}}{n_{1.7}}+\frac{I_{1.25}}{n_{1.25}}}{\frac{I_{3.68}}{n_{3.68}}}$$

were $I_{3.68}$ and $n_{3.68}$ are the integral and the number of protons of the CH_2 close to the OH end group, respectively.

•The DP of DS, BD and DLA-OH can be calculated taking into account the molar amount previously obtained with equation (SI 5):

$$\textbf{(SI 5)}\ [DP_n] = \frac{DP_{n+m}\cdot(Molar\ \%\ DS+Molar\ \%\ BD)}{100};\ [DP_m] = DP_{n+m} - DP_n$$

•M_n is obtained taking into account the DP, $Molar$ % and the $Molar\ mass$ of a hard segment composed by all the BD-DS units and a soft segment containing only DLA-OH as shown in equation (SI 6):

$$\textbf{(SI 6)}\ [M_n] = (DP_n \cdot Molar\ mass_{BD-DS}) + (DP_{n+m} \cdot Molar\ mass_{DLA-OH})$$

were $Molar\ mass_{BD-DS}$ is 172 g/mol and $Molar\ mass_{DLA-OH}$ is 538 g/mol.

•Finally, the experimental molar and weight % of hard and soft segments can be calculated as follows with equations **(SI 7-10)**:

$$\textbf{(SI 7)}\ [Molar\ \%\ HS] = \frac{\frac{I_{1.7}}{n_{1.7}}}{\frac{I_{1.25}}{n_{1.25}}+\frac{I_{1.7}}{n_{1.7}}}\ x100;\ \textbf{(SI 8)}\ [Molar\ \%\ SS] = 100 - Molar\ \%\ HS$$

$$\textbf{(SI 9)}\ [Weight\ \%\ HS] = \frac{Molar\ \%\ HS\cdot Molar\ mass_{HS}}{Molar\ \%\ HS\cdot Molar\ mass_{HS}+(100-Molar\ \%\ HS)\cdot Molar\ mass_{SS}}\ x100$$

$$(\mathbf{SI\ 10})\ [Weight\ \%\ SS] = 100 - Weight\ \%\ HS$$

Table SI.2. Theoretical Molar % and Weight % comparison with experimental values for PBS:DLS 70:30 and 50:50

	aMolar % BD:DS:DLA-OH	bMolar % BD:DS:DLA-OH	cMolar % HS:SS	dMolar % HS:SS	eWeight % HS:SS	fWeight % HS:SS	gMn (g/mol)
**PBS:DLS 70:30**	45:50:5	45:50:5	90:10	91:9	70:30	73:27	11590
**PBS:DLS 50:50**	50:39:11	50:41:9	79:21	82:18	50:50	55:45	27440

[a]Initial and [b]experimental (determined by ^1H NMR) feed molar ratio of monomers. [c]Initial and [d]experimental (determined by ^1H NMR) feed molar ratio between hard (HS) to soft segments (SS). [e]Initial and [f]experimental (determined by ^1H NMR) feed weight ratio between hard (HS) to soft segments (SS). [g]Determined by ^1H NMR.

SI.2 ^{13}C-NMR analysis

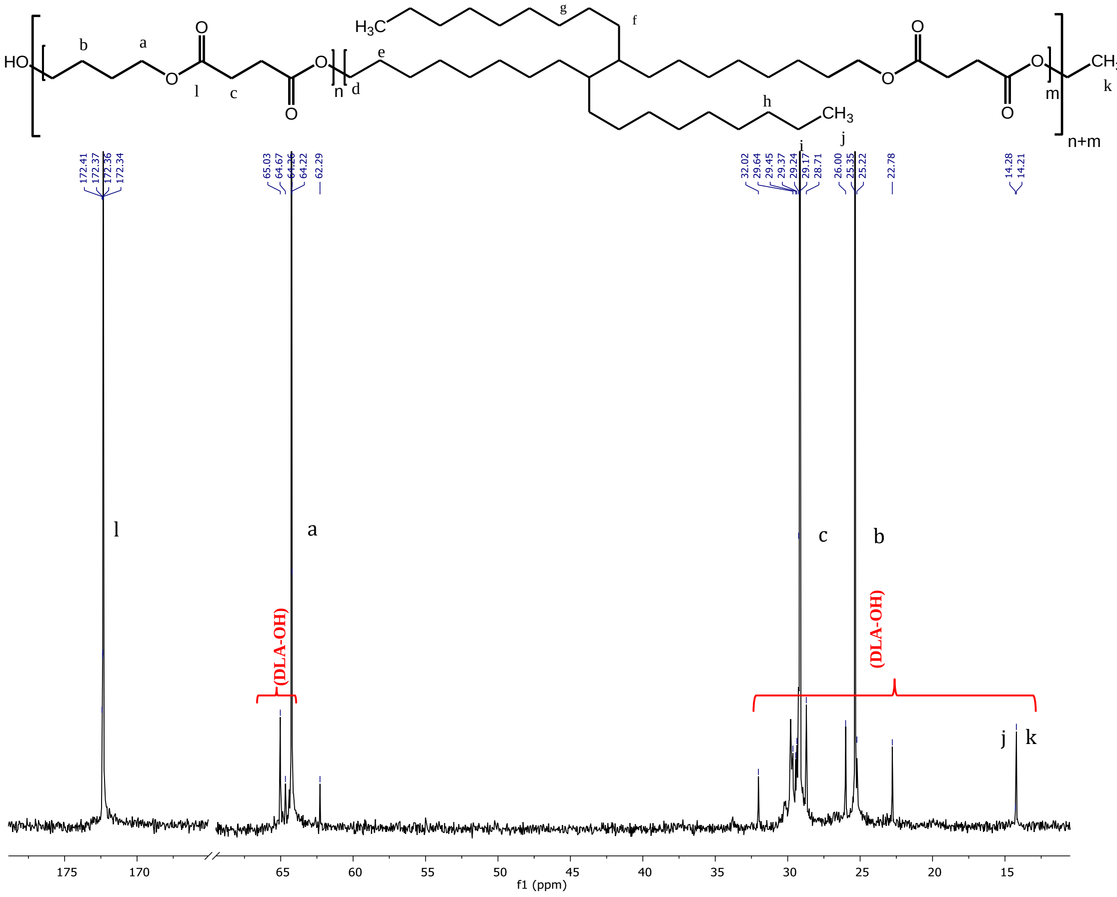

Figure S1. ^{13}C NMR of poly(butylene succinate-co-dilinoleic succinate) 70:30 copolyester.

Enzyme-catalyzed poly(butylene succinate-co-dilinoleic succinate) (PBS:DLS) 70:30 and 50:50 copolymers (^{13}C-NMR, CDCl$_3$, δ): 172.2 ppm (l; -*C*O-CH$_2$-CH$_2$-*C*O-, from DS), 64.3 ppm (a; -O-*C*H$_2$-CH$_2$-CH$_2$-*C*H$_2$-O-, from BD), 29.2 ppm (c; -CO-*C*H$_2$-*C*H$_2$-CO-, from DS), 25.3 ppm (b; -O-CH$_2$-*C*H$_2$-*C*H$_2$- CH$_2$-O-, from BD); 14.3 ppm (j; -CH$_2$-CH$_2$-*C*H$_3$, from DLA-OH), 22.7-31.9 ppm (d, e, f, g, h, i; -*C*H2- (aliphatic carbons), from DLA-OH).

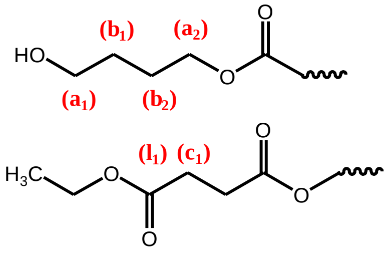

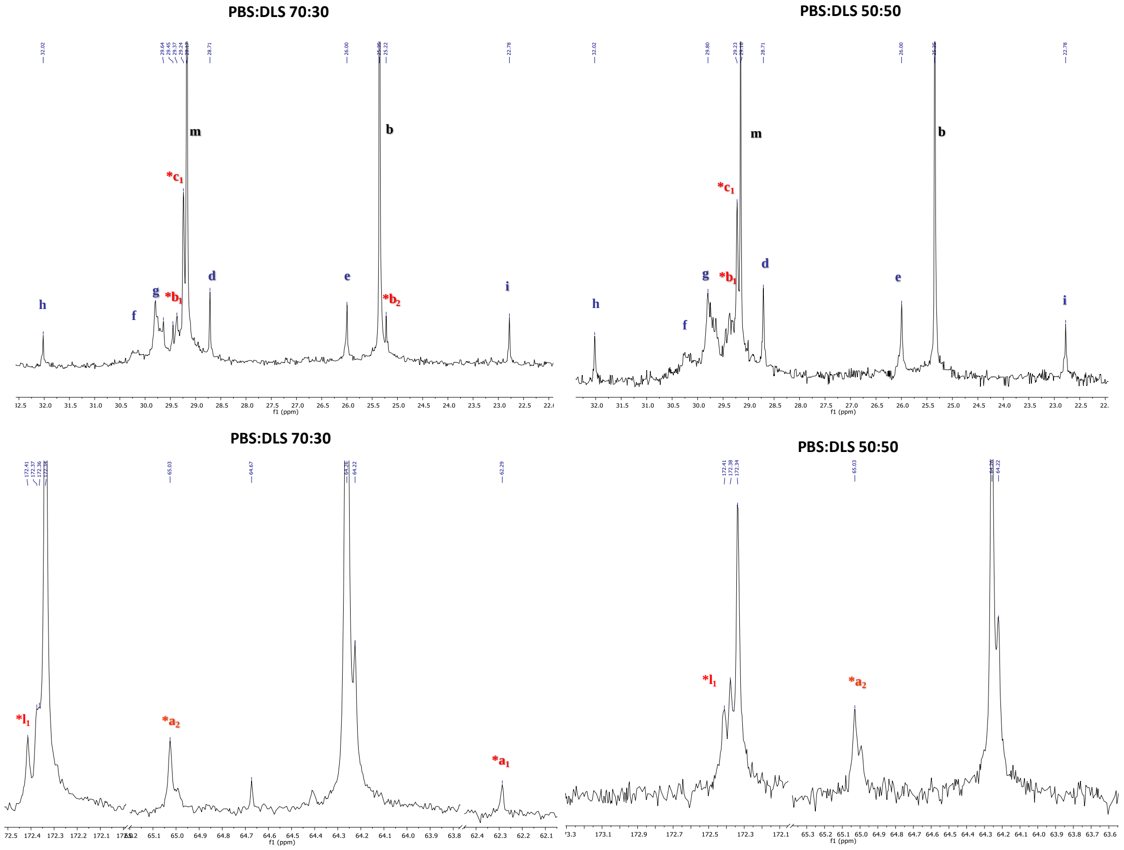

Figure S2. ^{13}C NMR regions showing DLA-OH peaks and BD and DS end-groups. Left, poly(butylene succinate-co-dilinoleic succinate) 70:30. Right, poly(butylene succinate-co-dilinoleic succinate) 50:50.

Low intensity resonances ascribed to BD end-groups at 65 ppm (a$_2$; HO-CH$_2$-CH$_2$-CH$_2$-*C*H$_2$-O-) and 62.3 ppm (a$_1$; HO-*C*H$_2$-CH$_2$-CH$_2$-CH$_2$-O-); low intensity resonances ascribed to ester end-groups from DS at 14.2 ppm (k; *C*H$_3$-CH$_2$-O-CO-), 29.16 (c$_1$; *C*H$_3$-CH$_2$-O-CO-CH$_2$-) and 172.3 (l$_1$; CH$_3$-CH$_2$-O-*C*O-). No end groups related to DLA-OH (*C*H$_2$-*C*H$_2$-OH) at 32.8 ppm and 63.0 ppm respectively were visible. In addition, a new resonance at 28.7 ppm (d; -O-*C*H$_2$-CH$_2$-) confirms the reaction of DLA-OH with DS

corroborating the structure proposed on the ^{1}H NMR analysis. In addition, the split of the carbonyl carbon peak at 172.3 ppm is related to the different environments due to a DS unit linked either to BD or DLA-OH on both sides, or BD on one side and DLA-OH on the other side.
